# Genetic polymorphism of COL1A1 gene and bone mineralisation in juvenile idiopathic arthritis

**DOI:** 10.1186/1546-0096-9-S1-P310

**Published:** 2011-09-14

**Authors:** MM Kostik, GS Demin, GV Stoyko, LA Scheplyagina, VI Larionova

**Affiliations:** 1 State Pediatric Medical Academy, Saint-Petersburg, Russian Federation; 2 “Gene” Ltd, Saint-Petersburg, Russian Federation; 3 Federal scientific and clinical center of pediatric haemotology, oncology and immunology, Moscow, Russian Federation

## Background

Bone mineralization disturbances are common in children with juvenile idiopathic arthritis (JIA) and depend on inflammation, medications, lack of motion and genetic factors.

## Aim

To evaluate the role of genetic markers in bone metabolism and mineralization in JIA children.

## Methods

We included 196 JIA children, 81 boys and 115 girls. Bone mineralization parameters were detected by dual-energy X-ray absorptiometry of lumbar spine L_1_-L_4_. Bone biochemical markers were osteocalcine, C-terminal telopeptides (CTT), parathyroid hormone (PTH), Ca, Ca^++^, P, total alkaline phosphatase (TAP) activity. We have detected Sp1 (rs1800012) and -1997G/T (rs1107946) polymorphisms in type I alpha-1 chain collagen gene (COL1A1).

## Results

We revealed gender differences in Sp1 genotype distribution in children with low bone mineral density (LBMD): boys had GG genotype in 89.5% and girls in 54.2% (p=0.03). In boys GG genotype presence increased LBMD – OR=2.96 (95%CI: 0.59-14.9) compare in girls in which GG presence decreased LBMD – OR=0.56 (95%CI: 0.36-2.7). Also, children carried T allele (GT and TT genotypes) despite on higher inflammatory parameters had better mineralization dates. In total group children with GG genotype had higher osteocalcine (111.0±56.1 ng\ml and 85.9±39.9 ng/ml in GT+TT, p=0.02) and CTT levels (1.22±0.45 ng\ml and 0.99±0.38 ng\ml in GT+TT, p=0.02). In children, who have not been treated with steroids GG genotype was associated with lower BMD-Zscore in boys (-1.24±0.14SD and 0.29±0.98SD in GT+TT, p=0.006) and lower height in girls (142.9±28.0 cm and 156.3±21.6 cm in GT+TT, p=0.025). In children with Tanner stage I GG genotype was associated with more rare LBMD (12.8% vs 36.4% in GT+TT, p=0.05) and with frequent LBMD in children with Tanner stage II-III (37.8% and 5.9% in GT+TT, p=0.01).

GG genotype of -1997G/T polymorphism was associated with lower Ca^++^ (1.1±0.11 mmol\l and 1.15±0.006 mmol/l in GT+TT, p=0.03), inorganic phosphate (1.67±0.16 mmol/l and 1.57±0.22 mmol/l in GT+TT, p=0.04) and osteocalcine level (82.3±18.4 ng/ml and 115.5±24.2 ng/ml, p=0.01) in children with Tanner stage II-III and lower BMD (0.84±0.14 g\cm^2^ and 0.91±0.1 in GT+TT, p=0.04) and lower BMD-Zscore (-1.275±1.25SD and -0.5±1.0SD in GT+TT, p=0.009)

## Conclusion

We have revealed different changes in mineralization and metabolism, associated with sex, Tanner stage and treatment due to COL1A1 gene polymorphisms in children.

